# Elevated levels of interleukin-18 are associated with several indices of general and visceral adiposity and insulin resistance in women with polycystic ovary syndrome

**DOI:** 10.20945/2359-3997000000442

**Published:** 2022-03-08

**Authors:** Plamena Kabakchieva, Antoaneta Gateva, Tsvetelina Velikova, Tsvetoslav Georgiev, Kyosuke Yamanishi, Haruki Okamura, Zdravko Kamenov

**Affiliations:** 1 Medical University of Sofia Medical Faculty Department of Internal Medicine Sofia Bulgaria Clinic of Endocrinology, University Hospital “Alexandrovska”, Department of Internal Medicine, Medical Faculty, Medical University of Sofia, Sofia, Bulgaria; 2 Naval Hospital – Varna Clinic of Internal Diseases Bulgaria Clinic of Internal Diseases, Naval Hospital – Varna, Military Medical Academy, Bulgaria; 3 Sofia University “St. Kliment Ohridski” University Hospital “Lozenetz” Laboratory of Clinical Immunology Bulgaria Laboratory of Clinical Immunology, University Hospital “Lozenetz”, Medical Faculty, Sofia University “St. Kliment Ohridski”, Sofia, Bulgaria; 4 Medical University – Varna Medical Faculty First Department of Internal Medicine Varna Bulgaria University Hospital “St. Marina”, First Department of Internal Medicine, Medical Faculty, Medical University – Varna, Varna, Bulgaria; 5 Hyogo College of Medicine Department of Psychoimmunology Japan Department of Psychoimmunology, Hyogo College of Medicine, Japan

**Keywords:** Polycystic ovary syndrome, interleukin-18, obesity, insulin resistance

## Abstract

**Objective:**

Our aim was to analyze levels of proinflammatory biomarker interleukin-18 (IL-18) in healthy controls and patients with polycystic ovary syndrome (PCOS) focusing on its association with obesity, clinical, hormonal, and metabolic characteristics.

**Subjects and methods:**

Fifty-eight patients with PCOS were enrolled in the study fulfilling the Rotterdam criteria and were matched for age, body mass index (BMI), and ethnicity with 30 healthy controls. Detailed anthropometric measurements, clinical investigations, hormonal and biochemical tests were obtained between the 3^rd^ and 5^th^ day of a menstrual cycle. A subanalysis of the PCOS group was performed separating patients into several groups according to a waist-to-height ratio (WHtR), insulin resistance (IR), and free androgen index (FAI). Serum IL-18 levels were measured using the ELISA method.

**Results:**

Levels of IL-18 were similar between PCOS patients and controls. IL-18 was higher in overweight/obese women compared to normal-weight women when analyzing all participants together and separately PCOS or controls group (p < 0.001, p < 0.001, p = 0.01, respectively). Additionally, IL-18 levels were higher in high-WHtR and IR subgroups compared to low-WHtR (p < 0.001) and non-IR PCOS women (p < 0.001). PCOS women with high FAI had greater serum IL-18 levels than normal-FAI patients (p = 0.002). Levels of IL-18 correlated positively with most of the anthropometric and metabolic parameters. In multiple linear regression, age, waist circumference, and fasting insulin were independently related factors with IL-18.

**Conclusion:**

Elevated levels of IL-18 were related to several indices of general and visceral adiposity and insulin resistance in PCOS.

## INTRODUCTION

Polycystic ovary syndrome (PCOS) is one of the most common endocrine disorders among women of reproductive age (1). PCOS is now perceived as both a reproductive syndrome, presenting clinically with hyperandrogenism, ovulatory dysfunction (including oligo-amenorrhea), polycystic ovaries, and infertility (1), and a metabolic disorder (2). Most women with PCOS have obesity and insulin resistance (IR) (3,4) that further increases the risk for type 2 diabetes, hypertension, dyslipidemia, nonalcoholic fatty liver disease (NAFLD), and metabolic syndrome (5-7).

All of the aforementioned and associated with PCOS conditions are considered chronic low-grade inflammatory disorders (8) where visceral adipose tissue is thought to play a central role (9). Being infiltrated with different immune cells (T-cells and macrophages) (10,11), fat tissue additionally produces various pro-inflammatory cytokines related to IR (12).

Interleukin-18 (IL-18) is a cytokine, discovered by Okamura and cols. in the late 20th century and initially was described as an interferon-gamma (IFNγ)-inducing factor (13). As a potent pro-inflammatory cytokine, elevated IL-18 levels were already observed in many low-grade inflammatory conditions such as obesity, metabolic syndrome (14,15), prediabetes (16), type 2 diabetes, latent autoimmune diabetes of the adults (LADA) (17), hypertension (18), and dyslipidemia (19).

Elevation of IL-18 (20-22) and other inflammatory biomarkers, such as C-reactive protein (CRP) (23,24), tumor necrosis factor (TNF-α), and interleukin-6 (25) was observed as well as in PCOS patients. Nevertheless, the inflammatory nature of PCOS is still debatable because of the great variability of reported findings; thus, there is insufficient evidence to conclusively resolve the issue (26,27). According to some authors, the independent association between PCOS and low-grade inflammation is not quite certain and it could be analyzed after clear stratification of participants according to body mass index (BMI) or other parameters, using clinically relevant cutoffs (27).

Our research aimed to determine the IL-18 levels in women with PCOS and compare them with healthy controls, to analyze its association with different markers for global and central adiposity, insulin resistance, and hyperandrogenism, and to determine which of those variables independently predict IL-18 levels.

## SUBJECTS AND METHODS

### Study population

Fifty-eight women with PCOS (25.9 ± 5.2 years) were consecutively enrolled in this cross-sectional observational study and matched for age, ethnicity, and BMI with 30 healthy controls (27.6 ± 5.2 years). All the PCOS patients were recruited in the Endocrinology Clinic of University Hospital “Alexandrovska”, Sofia, Bulgaria and met the Rotterdam criteria (28).

The control group consisted of healthy volunteers with a regular menstrual cycle without clinical or biochemical hyperandrogenism and a history of infertility. Exclusion criteria for all participants in the study were: pregnancy, hyperprolactinemia, thyroid dysfunction, premature ovarian failure, hypothalamic amenorrhea, congenital adrenal hyperplasia, androgen-producing tumors, Cushing's syndrome or disease, use of oral contraceptive, antiandrogen or insulin-sensitizing drugs (metformin, thiazolidinediones) for the last 3 months. The study protocol was approved by the Research Ethics Committee of the Medical University of Sofia, Bulgaria with approval protocol number 14/22.05.2020. Each participant signed written informed consent before recruitment.

### Anthropometric and clinical assessment

Anthropometric and clinical assessment was carried out by the same clinician to avoid an inter-observer error. All participants were evaluated for hirsutism (according to modified Ferriman-Gallwey [mFG] score), acne, alopecia (using the Ludwig visual scale), and acanthosis nigricans (AN). Detailed anthropometric measurements were done including the following parameters: height, weight, BMI (kg/m^2^), waist circumference (WC), hip circumference (HC), waist-to-hip ratio (WHR), and waist-to-height ratio (WHtR).

Obesity was defined as BMI ≥ 30 kg/m^2^, overweight as BMI between 25 and 29.9 kg/m^2^, and normal weight as BMI ≤ 24.9 kg/m^2^ and > 18,5 kg/m^2^ (29). Ultrasound pelvic investigation was performed by an experienced sonographer for assessment of polycystic morphology of the ovaries.

### Laboratory tests

After an overnight fasting state, blood samples were obtained for measurement of fasting plasma glucose (FPG), serum immunoreactive insulin (IRI), testosterone, dehydroepiandrosterone sulfate (DHEAS), androstenedione, 17-OH-progesterone, luteinizing hormone (LH), follicle stimulating hormone (FSH), and estradiol between the 3rd and 5th day of spontaneous or progesterone-induced menstrual bleeding. Additionally, fasting serum samples were collected, centrifuged, and stored in a freezer at −80 °C until its examination for assessment of IL-18 and sex hormone-binding globulin (SHBG). A standard oral glucose tolerance test (OGTT) was also performed with measurement of plasma glucose and IRI at 0, 60, and 120 min. The homeostatic model assessment of insulin resistance (HOMA-IR) was calculated using FPG and fasting insulin according to the following equation: HOMA-IR = FPG (mmol/L) x fasting insulin (mU/L)/22.5 (30). Testosterone was measured by employing electrochemiluminescence immunoassay (ECLIA) by analytics Elecsys 2010 with analytical sensitivity 0.069 nmol/L and CV 1.2-4.7%. SHBG was measured by enzyme-linked immunosorbent assay (ELISA), based on the principle of competitive binding with an analytical sensitivity of 0.23 nmol/L. The free androgen index (FAI) was calculated using the following equation: FAI=Testosterone (nmol/L)/SHBG (nmol/L) × 100.

All other hormonal and biochemical measurements were performed by standard techniques in the referent for Bulgaria Laboratory of the University hospital “Alexandrovska”. Serum IL-18 was measured by ELISA (Medical & Biological Laboratories Co., Ltd, Nagoya, Japan # code No 7620) with a detection limit of 12.5 pg/mL.

### PCOS subgrouping

A detailed subanalysis of the PCOS group was performed separating patients into several groups. According to WHtR, the patient group was divided into high-WHtR (WHtR ≥ 0.5; n = 30) and low-WHtR (WHtR < 0.5; n = 28) (31,32). Further, the PCOS group was divided into PCOS with IR (n = 25) and without IR (n = 33). IR was accepted when the homeostatic model assessment of IR (HOMA-IR) > 2.5 and/or peak insulin levels during an OGTT > 100 IU/mL (33). Finally, patients with PCOS were divided into two androgen groups according to FAI: normal FAI (FAI < 5, n = 39) and high FAI (FAI ≥ 5, n = 19) (34).

### Statistical analysis

Statistical analysis was conducted using the SPSS software version 21.0. The normality of data distribution was assessed with the Shapiro-Wilk test. Parametric tests (Independent Samples T-Test) were carried out when data were normally distributed and the hypotheses were presented as the difference between the mean values ± standard deviation (SD). In case of a skewed data distribution, a nonparametric Mann-Whitney U test was used to compare the variables and the hypotheses were presented as the difference between the medians (interquartile range [IQR]). Pearson and Spearman correlation analyses were used for normally and abnormally distributed data, respectively. Multiple regression analysis using a forward (probability for entry ≤ 0.98, probability for removal ≥ 0.99) and enter methods for the introduction of independent variables were performed to identify the main determinants of IL-18 levels among the variables. The sample size was calculated based on the IL-18 measurements from a previous study (35) using Sample Size Calculator Clinical Calc (https://clincalc.com/stats/samplesize.aspx). To obtain a power analysis with alpha 0.05 and power > 80%, the sample size resulted in at least 20 participants per group. For all comparisons, p < 0.05 was chosen as the level of significance at which the null hypothesis was rejected.

## RESULTS

### Comparison between PCOS and healthy women

The population in our study consisted of 58 PCOS patients and 30 healthy women who were matched for age, BMI, and ethnicity. Women with PCOS had significantly higher levels of testosterone, DHEAS, androstenedione, 17-OH-progesterone, FAI, and LH/FSH ratio compared to controls. Expectedly, healthy controls were less hirsute than patients. All other clinical, anthropometric, and metabolic parameters were similar between the two groups. IL-18 levels were also similar between patients and healthy controls (Table 1).

**Table 1:** Anthropometric, clinical, and laboratory characteristics in PCOS patients and controls

Variable	PCOS (n = 58)	Controls (n = 30)	P-value
Age (years)	25.9 ± 5.2	27.6 ± 5.2	0.102
Height (cm)	164 (160; 168)	165 (160; 169)	0.798
Weight (kg)	73 (58; 87)	70 (59.5; 97.3)	0.711
BMI (kg/m^2^)	27.7 ± 7.3	29.3 ± 7.4	0.738
WC (cm)	83.5 (76; 101.3)	84 (74; 96.8)	0.672
HC (cm)	100.5 (92; 110)	102 (92.8; 110.3)	0.724
WHR	0.84 (0.77; 0.96)	0.81 (0.76; 0.90)	0.296
WHtR	50.4 (45.3; 63.6)	48.7 (44.9; 57.4)	0.391
AN	21/58	6/30	0.147
Acne	14/58	6/30	0.791
mFG score	8 (4; 14)	3.5 (1; 6.3)	**<0.001**
FPG (mmol/L)	5.0 (4.8; 5.3)	5.05 (4.8; 5.2)	0.621
OGTT 60' glucose (mmol/L)	6.8 (5.1; 9.0)	6.7 (5.1; 8.0)	0.788
OGTT 120' glucose (mmol/L)	5.5 (4.8; 6.5)	5.5 (4.8; 6.3)	0.788
Fasting IRI (mU/L)	9.4 (5.9; 16.8)	9.3 (6.0; 14.4)	0.996
OGTT 60' IRI (mU/L)	80.9 (45.8; 125.9)	61.9 (30.9; 124.3)	0.379
OGTT 120' IRI (mU/L)	42.3 (23.4; 85)	30.1 (17.2; 49)	0.058
HOMA-IR	2.15 (1.25; 3.74)	2.18 (1.28; 3.27)	0.982
LH (mU/mL)	6.6 (4.7; 8.5)	5.8 (4.1; 8.1)	0.214
FSH (mU/mL)	5.1 (4.3; 6.1)	5.5 (4.8; 6.5)	0.333
LH/FSH ratio	1.2 (1.0; 1.8)	1.0 (0.8; 1.2)	**0.023**
Estradiol (pmol/L)	133.7 (96.9; 193.8)	147.2 (104.8; 190.3)	0.768
Testosterone (nmol/L)	1.6 (1.1; 2.0)	0.9 (0.7; 1.1)	**<0.001**
DHEAS (mcmol/L)	8.9 (6.5; 11.6)	5.5 (4.2; 7.5)	**<0.001**
Androstenedione (ng/mL)	4.4 (3.0; 5.5)	2.3 (1.5; 2.7)	**<0.001**
17-OH-progesterone (ng/mL)	1.6 (1.3; 1.9)	1.2 (1.0; 1.3)	**<0.001**
FAI	3.4 (1.7; 6.8)	1.5 (1.0;3.1)	**0.002**
SHBG (nmol/L)	45.1 (28.6; 79.1)	54.0 (28.3; 80.5)	0.544
IL-18 (pg/mL)	211.8 (134.6; 308.3)	249.8 (179.9; 367.1)	0.081

AN: acanthosis nigricans; BMI: body mass index; DHEAS: dehydroepiandrosterone sulfate; FAI: free androgen index; FPG: fasting plasma glucose; FSH: follicle stimulating hormone; HOMA-IR: homeostatic model assessment of insulin resistance; HC: height circumference; IRI: immunoreactive insulin; IL-18: interleukin-18; LH: luteinizing hormone; mFG score: modified Ferriman-Gallwey score; OGTT: oral glucose tolerance test; SHBG: sex hormone-binding globulin; WHR: waist-to-hip ratio; WHtR: waist-to-height ratio; WC: waist circumference.

Normally distributed data are presented by mean (±SD). Skewed distributed data are presented as median (25%; 75%). Categorical values are presented as a proportion of the total number (n/N).

### Comparison between overweight/obese and normal-weight women in the groups

**Figure 1:**
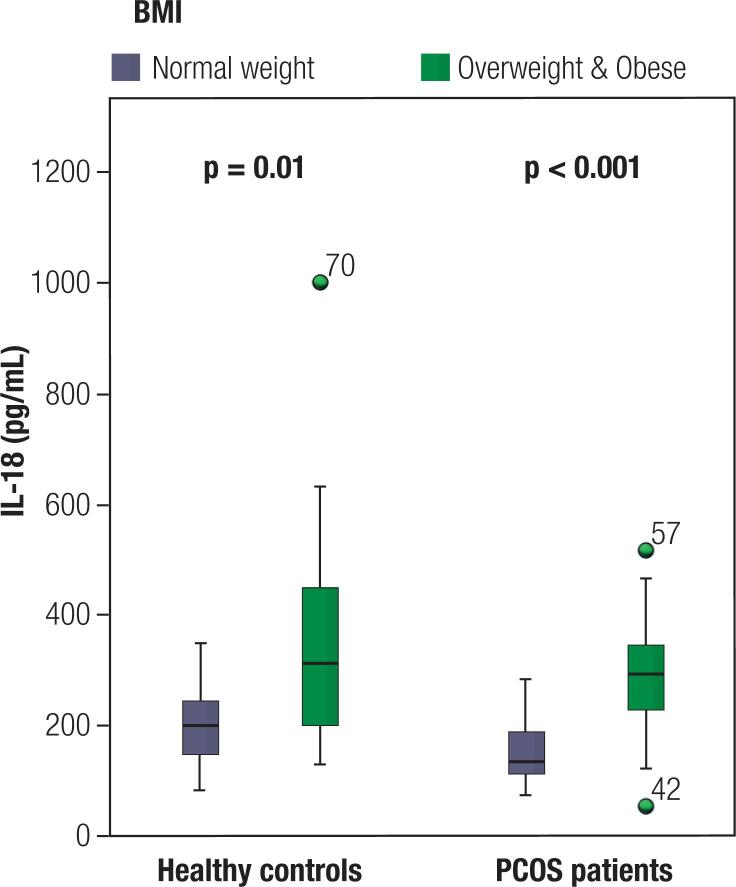
Clustering IL-18 levels in the study population. Overweight/obese women had higher serum IL-18 levels than normal-weight women in both healthy and PCOS patients together and separately. No difference in IL-18 levels was found between overweight/obese patients (n = 33) and overweight/obese controls (n = 17) and between normal-weight patients (n = 25) and normal-weight controls (n = 13).

When all participants were taken into account (n = 88), IL-18 levels were higher in overweight/obese women (n = 50) compared to normal-weight women (n = 38) (300.8 [211.4; 357] *vs.* 177.5 [114.5; 210.9], p < 0.001) and in both groups separately – control group (313.5 [199.6; 461.6] *vs.* 202.5 [132.3; 249.8], p = 0.01) and PCOS group (295.4 [223.1; 344.3] vs. 135 [112.3; 192.3], p < 0.001) (Figure 1). When comparing IL-18 levels between overweight/obese PCOS women and overweight/obese healthy controls, they did not showed significant difference (313.5 [199.6; 41.6] *vs.* 295.4 [223.1; 344.3], p = 0.208). Similar results were seen when comparing normal-weight women in the two groups (202.5 [132.3; 249.8] *vs.* 135 [112.3; 192.3], p = 0.064).

### Subanalysis in the PCOS group

**Figure 2:**
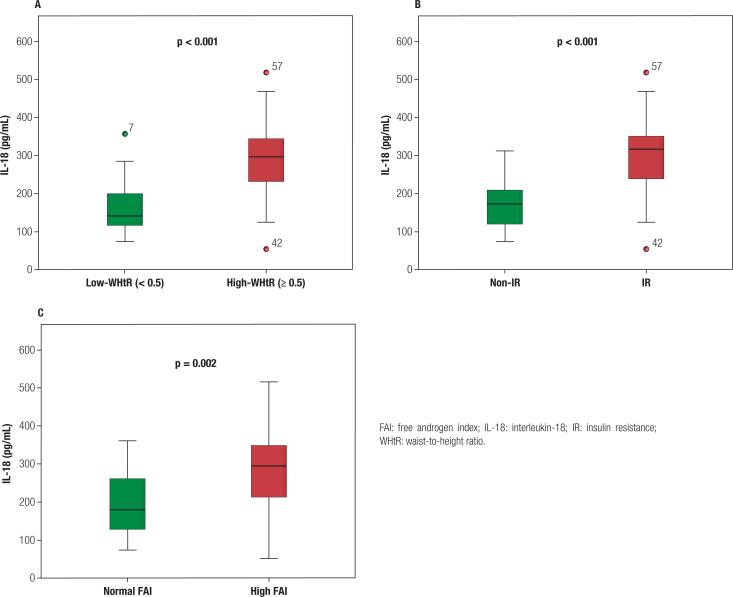
Clustering IL-18 levels in PCOS women. (**A**) Difference in serum IL-18 levels between low- WHtR (n = 28) and high-WHtR PCOS women (n = 30). (**B**) Difference in serum IL-18 levels between non-IR (n = 33) and IR (n = 25) PCOS women. (**C**) Difference in serum IL-18 levels between normal-FAI (n =39) and high-FAI (n = 19) PCOS women.

A detailed analysis in the PCOS group showed that high-WHtR patients had higher levels of IL-18, than low-WHtR participants (296.8 [227.5; 344.1] *vs.* 140.8 [115.7; 200.8], p < 0.001) (Figure 2A). IL-18 levels were significantly elevated in PCOS patients with IR, than those without IR (316.3 [237.1; 352.5] *vs.* 172.5 [119; 211.8], p < 0.001) (Figure 2B). IL-18 levels were also higher in PCOS women with high FAI than patients with normal FAI (298.3 [214.5; 355.3] *vs.* 181.1 [129.1; 262.4], p = 0.002) (Figure 2C).

### Correlations of IL-18 levels in the patients and controls

IL-18 levels correlated positively with almost all anthropometric and metabolic parameters in the PCOS group, presented in Table 2. In the control group, the serum levels of IL-18 did not show a correlation with hormonal and most of the anthropometric and metabolic parameters. The IL-18 levels correlated weakly and positively with WHtR (r = 0.381; p = 0.038) and OGTT 60' IRI (r = 0.434; p = 0.017).

**Table 2:** IL-18 correlation analysis in the PCOS group

Variable	r	p
BMI (kg/m^2^)	0.556	**<0.001**
Weight (kg)	0.544	**<0.001**
Height (cm)	- 0.089	0.505
WC (cm)	0.598	**<0.001**
HC (cm)	0.506	**<0.001**
WHR	0.420	**0.001**
WHtR	0.553	**<0.001**
FPG (mmol/L)	0.351	0.007
OGTT 60' glucose (mmol/L)	0.322	**0.015**
OGTT 120' glucose (mmol/L)	0.227	0.087
Fasting IRI (mU/L)	0.634	**<0.001**
OGTT 60' IRI (mU/L)	0.352	**0.008**
OGTT 120' IRI (mU/L)	0.356	**0.006**
HOMA-IR	0.639	**<0.001**
mFG score	0.286	**0.030**
LH (mU/mL)	- 0.169	0.206
FSH (mU/mL)	- 0.099	0.460
LH/FSH ratio	- 0.102	0.447
Estradiol (pmol/L)	0.073	0.584
Testosterone (nmol/L)	0.055	0.680
DHEAS (mcmol/L)	- 0.040	0.765
Androstenedione (ng/mL)	- 0.099	0.457
17-OH-progesterone (ng/mL)	0.010	0.940
FAI	0.232	0.080
SHBG (nmol/L)	- 0.240	0.070

BMI: body mass index; DHEAS: dehydroepiandrosterone sulfate; FAI: free androgen index; FPG: fasting plasma glucose; FSH: follicle stimulating hormone; HOMA-IR: homeostatic model assessment of insulin resistance; HC: height circumference; IRI: immunoreactive insulin; LH: luteinizing hormone; mFG score: modified Ferriman-Gallway score; OGTT: oral glucose tolerance test; SHBG: sex hormone-binding globulin; WHR: waist-to-hip ratio; WHtR: waist-to-height ratio; WC: waist circumference.

Pearson and Spearman correlation analysis (Rho) were used for normally and abnormally distributed data, respectively.

### Multiple linear regression in the PCOS group

**Figure 3:**
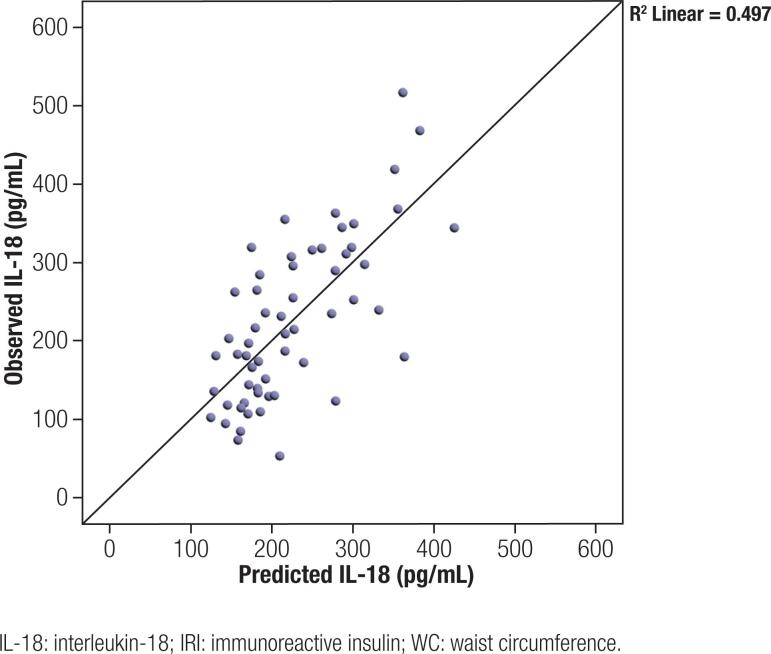
Scatterplot depicting the relationship between observed and predicted IL-18 values according to a linear regression model with IL-18 as a dependent variable and independent variables – age, WC, and fasting IRI. Approximately 50% of variations in IL-18 could be explained by these three variables.

Multiple linear regression was carried out to predict IL-18 levels from the reported demographic, anthropometric, and metabolic characteristics in the patient group. Using a forward stepwise regression model, only age, WC, and fasting IRI were selected for further analysis adding explanatory power. These variables significantly predicted IL-18, F(3, 55) = 17.817, p < 0.001, R2 = 0.497. All three variables added statistical significance to the prediction model, p < 0.05 (Figure 3).

## DISCUSSION

Metabolic abnormalities observed in women with PCOS are undoubtedly associated with chronic low-grade inflammation. Is PCOS *per se* however an inflammatory condition or the reported relationship between PCOS and low-grade inflammation is due to confounding factors? The present study aimed to address this question by comparing levels of IL-18, as a potent proinflammatory biomarker, in PCOS patients and healthy controls. Furthermore, we determined the variables which independently predicted IL-18 levels in PCOS patients.

Our study found no difference in IL-18 levels between PCOS patients and healthy controls. Intentionally, the two groups were matched for age, BMI, and ethnicity that probably predetermined similar values of most metabolic parameters. In contrast, PCOS patients were more hyperandrogenic and therefore more hirsute than controls. Notably, hyperandrogenism is the most important characteristic in the diagnostic criteria of PCOS according to the National Institute of Health (36) and the Androgen Excess and Polycystic Ovary Syndrome Society (37) and one of the three characteristics according to Rotterdam criteria (28).

Clinical research exploring the association between PCOS *per se* and IL-18 reported confusing results. Some of those studies showed that PCOS independently from obesity is linked to increased levels of IL-18 (20-22). However, Kaya and cols. (38) demonstrated that obesity explained IL-18 levels in PCOS women and impacted the significance in difference of the biomarker levels between patients and controls. Those data showed that obese PCOS women and obese healthy controls had similar levels of IL-18. Another study by Lindholm and cols. (39) demonstrated a lack of significance in IL-18 levels among the three groups in their analysis: lean PCOS women, overweight PCOS women, and overweight controls. More interestingly, when analyzing amounts of mRNA for inflammatory markers (including IL-18) in adipose tissue, the authors observed no differences between overweight PCOS patients and overweight controls, whereas inflammatory markers were higher compared to lean PCOS patients (39). A non-causal relation thus was previously suggested with PCOS and IL-18 levels because they both were linked to obesity as a confounding factor. We found similar results in line with recent studies that levels of IL-18 are increased in obese participants (20,22,38,39). In each of the three analyses (including all participants, healthy controls, or PCOS group, separately), overweight/obese women had significantly higher levels of IL-18 than normal-weight women. Importantly, PCOS status influenced the levels of IL-18 neither in overweight/obese participants nor in lean of them.

To enlighten the relation between inflammation and PCOS, the study was intended to contribute to the current knowledge and look from a different angle, taking into account relevant markers of global and central adiposity, insulin resistance, and hyperandrogenism. Importantly, the results of our study rather support the hypothesis that the association between PCOS *per se* and elevation of IL-18 levels is dependent on confounding factors such as obesity and insulin resistance which influence the cytokine levels resulting in low-grade inflammation in PCOS. Similar to prior research in the field (35), we confirmed that IL-18 levels were higher in PCOS patients with IR than without IR. In our study, however, greater interest was aroused in the investigation of PCOS patients divided according to WHtR and FAI using clinically relevant cutoffs, which was performed for the first time to the best of our knowledge. WHtR is an effective marker for the assessment of high metabolic and cardiovascular risk profiles (31,32). On the other hand, androgen excess is associated with a deteriorated metabolic profile (40) and FAI is a relevant parameter for the assessment of hyperandrogenism. The results from our two subanalyses with WHtR and FAI showed that IL-18 levels were significantly higher in patients with visceral obesity and hyperandrogenism. Further studies should assess the role of IL-18 as a predisposing factor for increased cardiovascular and additional metabolic risk among patients with PCOS.

IL-18 levels were correlated positively with several indices for general and visceral adiposity and most of the metabolic parameters associated with IR in the PCOS group. Although the hyperandrogenic PCOS patients had higher IL-18 levels than those with normal androgen levels, only mFG score showed a weak correlation with the interleukin levels. These results and the established link between IL-18 and deteriorated metabolic profile favor an assumption that visceral obesity and insulin resistance predispose to increased inflammation in PCOS patients rather than hyperandrogenism. In the multiple regression analysis, approximately 50% of variances in IL-18 levels were explained by age, WC, and fasting insulinemia.

The limitations of our study include a relatively small number of subjects and its cross-sectional design. Furthermore, we cannot determine if there is a cause-and-effect relationship between IL-18 levels and metabolic and cardiovascular risk in PCOS women. Larger and longitudinal studies are warranted to clarify such a relation.

In conclusion, in our study, levels of IL-18 were similar between patients with PCOS and healthy controls. IL-18 levels were related to several indices of general and visceral adiposity and insulin resistance in the PCOS group where age, waist circumference, and fasting insulinemia most closely explain serum amounts of this proinflammatory biomarker.
